# Risk Modulation by Electronic Cigarette Consumption in Cardiovascular Disease and Its Relevance in Cardiac Surgery—A Narrative Review

**DOI:** 10.3390/jcdd13090446

**Published:** 2026-09-08

**Authors:** Franziska Wittmann, Clemens Nebert, Teresa Ruthmeier, Daniel Zimpfer, Barbara Messner

**Affiliations:** 1 Department of Cardiac and Thoracic Aortic Surgery, Medical University of Vienna, 1090 Vienna, Austria; franziska.wittmann@meduniwien.ac.at (F.W.); teresa.ruthmeier@meduniwien.ac.at (T.R.); daniel.zimpfer@meduniwien.ac.at (D.Z.); 2 Division of Cardiac Surgery, Department of Surgery, Medical University of Graz, 8036 Graz, Austria; clemens.nebert@medunigraz.at

**Keywords:** electronic cigarettes, cardiovascular disease, cardiac surgery

## Abstract

The detrimental effect of cigarette smoking has become undeniable, based on decades of research. Twenty years ago, the electronic cigarette was introduced to the market and has since then been presented as the alleged healthy alternative. Clever marketing by the industry has led to a global rise in the number of users, especially adolescents and young adults. Clinicians and scholars have praised the electronic cigarette as a tool for tobacco smoke cessation, hoping for a reduction in burden on healthcare systems. Yet accumulating evidence suggests otherwise. Over the years, several in vitro and in vivo studies in animal models and humans have shown electronic cigarettes to be associated with pulmonary, cancerous and cardiovascular diseases. This review revisits the so-far available clinical evidence concerning cardiovascular diseases and discusses research gaps that still need to be addressed, specifically focusing on patients undergoing cardiac surgery.

## 1. Introduction

Electronic cigarette (e-cigarette) smoking has rapidly gained popularity as an alternative to traditional combustible tobacco products. Numerous types of e-cigarettes in a wide variety of flavors and with various additives are available on the market today. In the 1960s, tobacco companies started to develop an electronic counterpart to the conventional cigarette. Four decades later, the first e-cigarette was introduced to the Chinese market in 2003 and further development led to international expansion and evolvement of the product into several further “generations” [1]. Earlier models optically resembled the classical cigarette and were available as disposable or rechargeable devices. Newer products are mostly so-called pod systems and come in all shapes and forms. Current disposable e-cigarettes, including vapes, are compact, readily usable with different wattages and available in a multitude of different flavors. Pod systems consist of interchangeable cartridges—the “pods”—which are inserted into the e-cigarette device [2]. These pods are filled with liquid propylene glycol and glycerin as carrier substances for nicotine in varying concentrations and various flavors [3]. Most of these pods are disposable, while others can be refilled. Refillable products have been reported to entice the addition of other substances by the user themselves, such as tetrahydrocannabinol, and are thus posing additional health risks [4]. Simultaneously, regulations on the usage and composition of e-liquids are highly heterogeneous, with some countries not even having an age limit for buying e-cigarettes [5]. Insidiously, they are primarily attractive for young people—partly due to their sweet taste and colorful design, which are specifically aimed at teenagers and young adults. Data show that standardizing the packaging of e-cigarettes and restricting flavor variations could reduce the products’ appeal to adolescents, but not to adults [6].

The harmful effects of conventional smoking have been extensively studied, are well known, and are widely recognized by the public. This has led to decreasing numbers of cigarette smokers worldwide since the early 2000s [5]. At the same time, the use of e-cigarettes has been steadily increasing since its introduction to the market two decades ago, as they have a dubious reputation for being a healthier alternative. They are marketed as less harmful and as a useful tool to quit conventional cigarettes; the UK Government even launched an initiative in 2023 encouraging e-cigarette use as a measure to quit smoking [7]. However, this approach often results in dual use [8,9,10]. This is problematic in several dimensions, for example increased concentrations of heavy metals compared to conventional cigarette use only [11]. Survey data from the US in 2021 further highlighted that a considerable number of e-cigarette users were never smokers before [8]. Data regarding the long-term effects on the cardiovascular system are scarce. Yet epidemiological evidence increasingly links e-cigarettes to various cardiovascular diseases (CVDs), including cardiac arrhythmia, hypertension, acute coronary syndromes and heart failure [12]. E-cigarettes were introduced to the market a mere 20 years ago and have since then been ever-changing, making the generation of valid longitudinal data difficult [13]. The nicotine content of e-cigarettes has generally increased over the years and varies greatly among brands [14,15]. Frequent mislabeling of nicotine content further complicates evaluation of nicotine concentration-dependent effects on cardiovascular outcomes. Alarmingly, several studies detected significantly higher concentrations than indicated on packaging or even nicotine present in products labelled nicotine-free [16]. Furthermore, nicotine uptake varies depending on the length and volume of single inhalations, time in between puffs and device wattage. A higher wattage and longer puff duration result in higher nicotine levels [17]. Interestingly, e-cigarette users tend to have quite different smoking behavior compared to the average conventional smoker. Whereas never-before users tend to perform shorter puffs, experienced e-cigarette smokers are more likely to inhale longer, resulting in higher nicotine serum levels [18]. Of note, these observations have not been validated in larger cohorts. Another influencing factor is the protonation state of nicotine. Currently, un- and mono-protonated forms are in use—free-base nicotine and nicotine salts, respectively. They exhibit quite different chemical properties; yet data on which yields higher nicotine levels remain unclear [19].

Future research must aim to establish a better understanding of the effects of e-cigarette use, incorporating specific data on the diverse electronic systems available. Therefore, this review aims to summarize the existing literature on the effects on the cardiovascular system, with a particular focus on implications in cardiac surgery, and to generate a comprehensive overview of the knowledge gaps. Specifically, it will explore how e-cigarettes influence cardiac physiology, outcomes and recovery trajectories after cardiac surgery.

## 2. Methodology

The literature search was carried out between June and August 2026 and focused on the most recent data. We searched PubMed and Google Scholar using the search terms “electronic cigarette smoking” and “vaping” in combination with the following key words: cardiovascular disease, blood pressure, hypertension, hemodynamics, heart rate variability, surgery, cardiac surgery, cardiothoracic surgery, anesthesia, cardiopulmonary bypass, surgical outcomes, postoperative complications, wound healing, ventilation times, intensive care stay, oral health, nicotine-free and the term “electronic versus conventional cigarette smoking”. The focus of this review was available clinical data in humans; therefore, we mostly omitted in vitro studies and studies performed in animal models. Moreover, we conducted a search for already published reviews on e-cigarettes to avoid redundancy.

## 3. Results

### 3.1. E-Cigarette Smoking and Cardiovascular Diseases

The effects of e-cigarettes on the cardiovascular system are multifaceted. Several papers have been published over the years addressing such effects and multiple reviews are already available [20,21,22]. Hence, the present review will provide only a short overview. The influence of e-cigarettes on blood pressure and arterial stiffness has been investigated in several randomized clinical trials. Of note, experimental set-ups varied and cohorts were small, with only 12 to 16 participants. Two studies analyzed the effects in actively smoking healthy young adults. One included only women (n = 15) and observed no effect after e-cigarette use but significantly increased arterial stiffness after smoking a conventional cigarette [23]. However, in an all-male cohort (n = 12), both electronic and conventional cigarettes led to comparable increases in blood pressure and heart rate [24]. An earlier study including both sexes and active smokers (n = 15) reported similar outcomes and additionally included nicotine-free e-cigarettes, which did not lead to hemodynamic changes [25]. Two studies similar in set-up included non-smokers and both sexes. Whereas one study observed no significant hemodynamic changes [26], the other reported an increase in blood pressure and heart rate after e-cigarette use and no effect after nicotine-free e-cigarettes [27]. Moheimani et al. conducted two studies modeling acute and chronic use of e-cigarettes; those containing nicotine were associated with increased cardiac sympathetic nerve activity in both settings [28,29]. These studies imply that the effects on hemodynamics are primarily mediated by nicotine. Nicotine is a potent sympathomimetic, increasing heart rate, blood pressure and myocardial oxygen demand [12]. Importantly, e-cigarettes under certain conditions can induce higher serum nicotine levels compared to conventional cigarettes [17,18]. El-Mahdy et al. established a mouse model to mimic long-term smoking, comparing conventional to e-cigarette exposure with different concentrations of nicotine. They demonstrated that e-cigarette use caused time- and nicotine concentration-dependent induction of vascular dysfunction correlating with oxidative stress comparable to that induced by conventional cigarettes [30].

Several studies analyzing survey data are available. Large datasets such as the National Health Interview Survey in the U.S. were used to identify possible associations between e-cigarette use and the incidence of CVDs. Overall, studies reported conventional cigarette use and dual use, in particular, to be associated with a higher risk of CVD [31,32,33,34]. Critcher et al. analyzed survey data from 2014 to 2019 and observed an association of e-cigarettes and CVD only in patients who were former conventional smokers [35]. Recently published data analyzing the US National Health and Nutrition Examination Survey found hypertension to be associated with conventional cigarettes but only a trend was observed in e-cigarette users [36]. Another study combining survey data and clinical data from male railway workers in Shanghai reported a positive relationship between e-cigarette use and increased blood pressure [37]. However, their conclusions are built on rather weak data and must be viewed critically. Another study using data from more than 10,000 participants of the Population Assessment of Tobacco and Health Study identified conventional cigarette smoking to be associated with higher rates of myocardial infarction and stroke, with no correlation with e-cigarette smoking or dual use [38]. Of note, only 1% of participants were dual users, and there were even less exclusive e-cigarette users compared to 14.2% of conventional smokers. All of these studies have substantial limitations, mostly due to possible self-reporting bias and missing data on smoking length and intensity, often without differentiation between habitual and heavy smoking. Pre-clinical data are mostly available in rodent models and would exceed the scope of this review. Sgai et al. present a good overview [39].

Beyond nicotine, other toxic components such as carbonyl and volatile organic compounds and heavy metals identified in e-cigarette aerosols have been implicated in the progression of atherosclerosis [12,27,40]. Specifically for heavy metals such as cadmium and lead, strong data are available on their role in CVDs [41,42]. Carbonyl compounds such as formaldehyde have been shown to exhibit cardiotoxicity [43]. However, data on relevant exposure of carbonyl groups via e-cigarette smoking are controversial. Some studies reported significantly higher levels of formaldehyde induced by e-cigarettes compared to conventional cigarettes [44,45], whereas others reported lower exposure levels [46,47]. Yet substantial work from Jensen et al. has shown not only relevant concentrations of formaldehyde, but also additional possibly toxic compounds in e-cigarette smoke [48,49]. These data highlight the increased risk of CVDs from e-cigarettes independent of nicotine.

### 3.2. E-Cigarette Smoking and the Surgical Patient

#### 3.2.1. Perioperative Complications

Conventional cigarette smoking has been established as independent risk factor for perioperative complications, one being impaired wound healing. A randomized controlled trial carried out prior to the introduction of e-cigarettes identified tobacco smoking to significantly increase the risk of wound infections [50]. Accumulating evidence suggests that e-cigarettes do not differ in this regard. Kennedy et al. demonstrated in an animal model that e-cigarette smoke exposure negatively affects wound healing [51]. Clinical data from human studies are still limited. One prospective clinical study observed prolonged oral wound healing in e-cigarette smokers. They had higher rates of bleeding and swelling and exhibited impairment of keratinization of the epithelium after punch biopsies compared to never-smokers [52]. Based on the general belief that tobacco alternatives are healthier, multiple studies retrospectively analyzed different orthopedic procedures, evaluating whether available alternatives to conventional cigarettes meant less postoperative complications. Yet combined use of e-cigarettes containing nicotine, nicotine pouches or other nicotine delivery products was associated with an increased risk of postoperative complications, especially concerning early wound healing [53,54,55,56]. Comparable results were obtained in patients undergoing breast surgery [57]. However, none of these studies specifically addressed e-cigarettes or included a control group with nicotine-free e-cigarettes.

A possible association between e-cigarette exposure and thromboembolic events has been suggested by several in vitro studies and animal models. Components in e-cigarette smoke have been shown to induce platelet activation, the generation of oxidative stress and thus a pro-thrombotic milieu [58,59]. Nocella et al. confirmed the effect on platelet activation in humans [60]. The authors performed a crossover single-blind study to compare effects on platelet function prompted by smoking an e-cigarette or a conventional cigarette in active and non-smokers. Smoking a conventional and a nicotine-containing e-cigarette both acutely increased markers of platelet activation; changes were more pronounced in active smokers [60]. A similar study also reported elevated markers related to platelet activation after nicotine-containing e-cigarette use and to a limited extent after nicotine-free e-cigarette smoking [61]. These results were confirmed by Lyytinen et al. in healthy volunteers who were occasional smokers. They observed enhanced platelet formation after e-cigarette exposure; no relevant pro-thrombotic changes were seen using nicotine-free liquids [62].

#### 3.2.2. Implications for Anesthesiologists

Some scholars have raised the question of whether patients using e-cigarettes require special considerations during and after anesthesia [63,64]. The most obvious consideration regards possible implications for mechanical ventilation. However, data are very limited. A recent study by Saab et al. hypothesized that patients using e-cigarettes might be at risk of postoperative hypoxemia, longer ventilation times and pulmonary complications. They performed a retrospective cohort study but observed no differences between e-cigarette users and non-users [65]. Of note, concurrent or former conventional smoking was not clearly separated. Despite claiming to have statistically accounted for that confounder, both groups contained patients with conventional cigarette use, possibly accounting for significant bias. Moreover, the data do not clearly differentiate between e-cigarettes with or without nicotine. Impairment of respiratory function would certainly affect safe administration of anesthesia. An earlier small observational cohort study performed functional magnetic resonance imaging (MRI) and analyzed lung perfusion prior to and after smoking, comparing conventional to e-cigarettes. Conventional cigarette smokers exhibited a reduction in local lung perfusion after exposure. Contrary to that, lung perfusion in e-cigarette users containing nicotine significantly increased after smoking, whereas no changes were detected in subjects using nicotine-free e-cigarettes [66]. Comparable results have been published in 2020 by Kizhakke Puliyakote et al. who used MRI imaging to investigate changes in ventilation and perfusion provoked by nicotine-containing e-cigarettes. They similarily observed an increase in lung perfusion and significantly increased ventilation–perfusion heterogeneity. The authors further described that ventilation–perfusion matching was already abnormal at baseline in these young, seeminlgy healthy volunteers and that these changes resemble data from patients with early-stage chronic obstructive pulmonary disease [67]. However, no data are available on the long-term effects of these observed short-term changes in ventilation and perfusion. Kerr et al. performed a prospective randomized cross-over study in healthy male habitual tobacco smokers. They compared spirometry analyses prior to and after smoking conventional and e-cigarettes containing nicotine. Interestingly, they observed no significant changes in respiratory function but a reduction in peak expiratory flow after smoking an e-cigarette. The authors suggested this to be indicative of an irritation reaction of the airways, caused by propylene glycol in the e-cigarette aerosol [68]. Of note, a reduction in peak expiratory flow can also be suggestive of constrictive lung disease.

Moreover, there are some small-cohort studies reporting pulmonary inflammation and impairment of gas exchange in the alveoli in relation to e-cigarette smoking [69,70]. Yet these studies produced differing results concerning nicotine-free e-cigarettes; one study observed these effects after the usage of nicotine-free e-cigarettes [69], while another reported markers of an inflammatory response only after the usage of nicotine-containing e-cigarettes [70].

#### 3.2.3. Oral Health

Tobacco smoking has been clearly linked to deleterious effects on the oral cavity, such as gingivitis, caries and cancer [71]. Evidence on e-cigarette smoking on that matter is scarce. The first clinical study was published 10 years ago and examined gingival changes in conventional smokers switching to e-cigarettes for two weeks. They observed significantly higher rates of gingival bleeding upon probing, similar to previously described effects of tobacco cessation [72]. Counterintuitively, more bleeding is interpreted as the sustained inhibitory effect of tobacco smoking on the local inflammatory response and is thus a beneficial effect in this specific setting [73]. Other studies also reported improved oral health in smokers who converted to e-cigarettes compared to active conventional smokers [72,74], with comparable results from another including e-cigarette smokers who were primarily never-smokers [75]. Similar effects of smoking were observed concerning variables determining dental implant success, with worse outcomes in conventional smokers [76]. Yet, in all of these studies, e-cigarette users still had worse oral health than never-smokers. Bardellini et al. reported higher incidences of oral mucosal lesions in active e-cigarette versus former conventional smokers; however, they did not compare the results to active conventional smokers or never-smokers [77]. Data available on susceptibly to dental caries related to e-cigarette smoking are scarce. Ghazali et al. observed no differences in caries incidence when comparing conventional, electronic and non-smokers in a prospective cohort [78]. A large cross-sectional study, analyzing U.S. survey data, reported an increased risk of untreated dental cavities in e-cigarette smokers compared to never-smokers [79]. In vitro studies were able to show that exposure to e-cigarettes alters the oral microbiome, leading to conditions more susceptible to dental caries [80]. These mechanisms seem to be associated with the sugar content in e-cigarettes containing different added flavors [81].

## 4. Discussion

Two decades after the introduction of e-cigarettes, we are still lacking valid, reliable and comprehensive data describing the effects on the cardiovascular system. Thorough scanning of available reviews on e-cigarette smoking and its clinical implications in CVDs revealed that most keep citing the same publications. Substantial work has already been performed in animal models and cell biological studies. Clinical studies in humans, however, were often of questionable study design and associated with a high risk of bias [82]. Smoking status is heterogeneously defined across different studies. More often than not, the effects of electronic and conventional cigarette smoking are indistinguishable due to insufficient separation of former conventional smoking as a confounder and its potential long-term effects. Furthermore, study cohorts are rather small across the board; large-scale longitudinal clinical studies are missing. Large datasets are available from different national and international surveys. One group postulated an association of e-cigarette smoking with myocardial infarction based on data from the National Health Interview Surveys [83]. However, Critcher et al. critically re-examined their data and identified severe bias of these results. They suggest that the observed elevated risk of myocardial infarction relies solely on conventional cigarette smoking and highlight one major bias of these survey data—that it is often unclear whether e-cigarette use was initiated prior to or after the event [35]. At the end of the day, we have still very limited data.

Even less reliable data are available on the implications of e-cigarette smoking in the perioperative setting. Interestingly, several cohort studies are available on orthopedic and plastic surgery interventions linking alternative nicotine delivery products, including e-cigarettes, to impeded wound healing and higher rates of other postoperative complications such as thromboembolic events. No data are available in patients undergoing cardiac surgery. We hypothesize that the patient population in need of cardiac surgery might currently still have a lower percentage of active e-cigarette users as they are generally older compared to orthopedic and plastic surgery patients. However, due to increasing numbers of e-cigarette smokers, higher rates of postoperative complications could become relevant in cardiac surgery in the future.

The described negative effects on wound healing are likely nicotine-related, based on its vasoconstrictive effect [84]. Available clinical data do not include nicotine-free e-cigarettes. However, a study performed in a rodent model, which investigated the effects on wound healing in a comparison of e-cigarettes with different nicotine concentrations, observed slower wound healing in response to nicotine-free exposure similar to nicotine-containing smoke [85]. Furthermore, Pitzer et al. was able to demonstrate vasoconstrictive effects of nicotine-free e-cigarettes in their rodent model [86]. An in vitro study analyzing the effects of nicotine-free e-cigarette smoke extract on human lung fibroblasts indicates induction of inflammation and impairment of repair mechanisms in connection with wound healing processes [87]. The results from these pre-clinical studies indicate deleterious effects of e-cigarette smoking on wound healing independent of nicotine.

Data from in vitro and animal studies indicate pro-thrombotic effects of e-cigarette smoking [58,59]. Several small-cohort studies validated these results for nicotine-containing e-cigarettes in humans; an observed effect in nicotine-free products is inconsistent in the literature [60,61,62]. Cardiopulmonary bypass, necessary in most cardiac surgeries, induces coagulopathy associated with increased postoperative bleeding risk [88]. Therefore, a possibly pro-thrombotic tendency of e-cigarette smokers might not be relevant in these cases. However, the numbers of off-pump interventions in cardiac surgery are growing and could be associated with a higher risk of postoperative thromboembolic events in patients actively using e-cigarettes.

Studies indicating possible implications on patient care in the perioperative setting by the anesthesiologist are scarce. There are comments and some reviews available that suggest that anesthesiologists need to regard the patients’ history of e-cigarette smoking, yet these are without clear, unbiased data on the topic [63,64,65]. A few small clinical trials report significant changes in respiratory function and ventilation–perfusion mismatch in association with e-cigarette use [66,67,68]. One of these reported ventilation–perfusion mismatches to be present in their cohort of healthy, young volunteers who were occasional e-cigarette users. The authors described these observations as similar to changes usually seen in patients with early-stage chronic obstructive pulmonary disease [67]. These findings, in combination with evidence linked to pulmonary inflammation and altered alveoli gas exchange [69,70], indicate detrimental effects on pulmonary function in young, healthy users. We hypothesize that the described altered pulmonary physiology has the potential to interfere in safe mechanical ventilation and should be evaluated in further studies.

Evidence further suggests that e-cigarette smoking is associated with decreased oral health. The reported higher risk of gingivitis and dental cavities specifically [79] could impact postoperative trajectories in cardiac surgery. Oral infections are one of the most common causes for endocarditis [89]. Moreover, a recent in vitro study showed a modulating effect of flavored e-cigarettes on an oral pathogen commonly associated with endocarditis, potentially increasing its pathogenicity [90]. These data could indicate a higher risk of postoperative endocarditis after cardiac surgery, specifically valve surgery. Conventional cigarette smoking has long been a recognized independent risk factor for morbidity and mortality in patients undergoing cardiac surgery [91]. Given the growing evidence on the comparable effects of e-cigarettes to conventional smoking, it should be equally considered a risk factor.

## 5. Limitations

Since this review is of a non-systematic nature, the inclusion of studies in this work is not free of bias. Furthermore, the available data are limited, the study design of the available studies is heterogeneous and all studies included rather small patient cohorts.

## 6. Conclusions and Future Perspectives

The number of e-cigarette smokers is rapidly growing and represents a global phenomenon. The largest fraction consists of adolescents and young adults, who are actively targeted by marketing campaigns. These individuals might have never picked up a conventional cigarette due to immense efforts in recent decades to tackle the “tobacco epidemic”. At the same time, research is lagging behind in identifying the long-term consequences on public health. We are in desperate need of prospective large-cohort studies that allow us to gain comprehensive knowledge on the long-term effects of e-cigarette smoking. Strict separation from conventional tobacco as a confounder is necessary, as are efforts to identify and differentiate between the different available types of e-cigarettes and their specific harmful constituents, including nicotine-free products. Yet we have to acknowledge the difficulties that current research is facing. Weak regulations on e-cigarette constituents allow for a multitude of varying compositions that are ever-changing and difficult to unify. In comparison to the conventional cigarette, e-cigarettes are a heterogeneous amalgam complicating the generation of comprehensible data.

As a scientific community, we must come together to tackle this problem more strategically. The main goal should be the creation of standardized protocols to generate comparable data allowing for overarching conclusions. Only then can compelling data be provided and aid us in containing the on-going “e-cigarette epidemic”.

## Data Availability

No new data were created or analyzed in this study. Data sharing is not applicable to this article.

## Abbreviations

The following abbreviations are used in this manuscript:

E-cigarette	Electronic cigarette
CVD	Cardiovascular disease
MRI	Magnetic resonance imaging
